# Supplementary material for: Rapid Characterization and Discovery of Chemical Markers for Discrimination of Xanthii Fructus by Gas Chromatography Coupled to Mass Spectrometry
Source: Molecules. 2019 Nov 11;24(22):4079. doi: 10.3390/molecules24224079 (PMC6891722; doi:10.3390/molecules24224079)
Supplement: Supplementary file 1 [file molecules-24-04079-s001.pdf]

Table S1. Potential candidates for the discrimination between *X. canadense M* and *X. sibiricum PW* obtained by VOCs profiles.

No.	RT (min)	Compounds	Quantifying ion(m/z)	VIP value	*X. canadense M* Area RSD (%)	*X. sibiricum PW* Area RSD (%)
**Acid**						
1	16.17	Hexanoic acid	60	14.2	13.7	8.51
**Alcohol**						
2	11.43	7-Octen-4-ol	57	3.9	16.6	12.3
3	13.06	2,3-Butanediol	45	1.7	6.9	14.1
4	13.55	2-Octen-1-ol, (E)-	57	1.7	18.0	16.6
**Aldehyde**						
5	12.08	2,4-Heptadienal, (E,E)-	81	1.4	7.3	11.6
6	16.55	Benzyl alcohol	79	1.3	13.2	8.9
7	16.94	Benzeneethanol*	91	2.9	14.7	10.5
8	12.50	Benzaldehyde*	106	1.4	13.8	6.8
9	13.95	Benzeneacetaldehyde	91	2.2	13.7	8.5
10	18.10	1H-Pyrrole-2-carboxaldehyde*	66	1.2	12.9	8.8
**Cycloalkane**						
11	17.94	Decalin, anti-1-methyl-, cis-	82	3.1	18.2	6.6
**Alkene**						
12	11.11	(3E)-3-ETHYL-2-METHYL-1,3-HEXADIENE	67	1.6	12.4	9.6
13	15.44	2-Methyl-2-heptene	69	1.0	15.6	8.3
**Ester**						
14	19.84	Hexadecanoic acid, methyl ester	74	1.1	20.5	10.2
**Ketone**						
15	5.79	1-Penten-3-one, 2-methyl-	69	1.1	21.7	7.7
16	10.93	3-Octen-2-one*	55	2.3	13.9	9.8
**Lactone**						
17	13.61	γ-Valerolactone	56	1.2	11.4	10.0
18	13.83	Butyrolactone*	86	1.2	11.0	7.8
19	14.71	γ-Caprolactone*	85	3.6	12.3	9.7
20	15.77	δ-Hexalactone*	70	1.8	12.5	9.8
21	18.14	Pantolactone*	71	2.3	9.3	9.3
22	18.20	γ-Octalactone	85	5.2	16.8	7.8
23	18.84	Carvotanacetone*	59	2.4	9.7	10.4
24	21.28	2(4H)-Benzofuranone, 5,6,7,7a-tetrahydro-4,4,7a-trimethyl-, (R)-	111	1.5	11.4	9.8
25	21.30	2-BENZOFURAN-1(3H)-ONE	105	1.2	10.9	11.9
**Imide**						
26	20.39	2-Ethyl-3-methylmaleimide	139	1.2	9.7	9.4
27	22.12	Succinimide	99	1.5	11.1	10.3

Table S2. Potential candidates for the discrimination of polar metabolites betwee-n *X. canade nse M* and *X. sibiricum PW* obtained by GC-TOF MS

No.	RT (min)	Compound name	Quantifying ion(m/z)	VIP value	*X. canadense M* Area RSD (%)	*X. sibiricum PW* Area RSD (%)
**Alcohol**						
1	7.1	Ethylene glycol*	147	1.6	10.4	9.8
2	14.7	Mesoerythritol	217	1.2	2.9	5.1
3	17.2	L-(-)-Arabitol*	73	10.3	14.9	6.0
4	19.4	D-Mannitol*	319	3.7	12.6	6.2
5	20.3	Scyllo-inositol*	318	4.4	4.3	14.3
6	20.4	L-Fucitol	117	1.0	3.8	8.1
**Acid**						
7	7.2	N,N-Dimethylglycine	58	1.7	16.4	11.8
8	9.7	3-Hydroxypropanoic acid	147	1.3	6.94	7.9
9	12.2	Succinic acid*	247	1.6	3.64	4.1
10	12.4	D-Glyceric acid*	189	1.7	4.8	9.1
11	12.7	Fumaric acid*	245	1.2	5.2	5.6
12	14.5	Malic acid*	73	10.1	7.6	11.2
13	15.3	Erythronic acid	73	3.5	5.9	13.5
14	18.1	Azelaic acid*	55	1.1	5.8	7.7
15	20.1	Gluconic acid*	333	1.3	6.7	19.1
**Ester**						
16	10.1	L-Proline, 1-methyl-, methyl ester	84	1.6	23.1	8.1
**Ketone**						
17	18.2	2,4-Imidazolidinedione	93	1.0	12.3	15.9
**Monosaccharide**						
18	19.3	d-Mannose	319	2.4	4.1	6.3
19	32.3	D-Psicofuranose*	230	1.2	13.4	15.5

* Quantitative analysis was performed using their corresponding authentic standards
